# Update on Ppar**γ** and Nonalcoholic Fatty Liver Disease

**DOI:** 10.1155/2012/912351

**Published:** 2012-08-16

**Authors:** Gene P. Ables

**Affiliations:** Animal Science Laboratory, Orentreich Foundation for the Advancement of Science, 855 Route 301, Cold Spring, NY 10516, USA

## Abstract

Nonalcoholic fatty liver disease (NAFLD) is the most common initial presentation of obesity and insulin resistance. Uninterrupted progression of hepatic lipid accumulation often leads to fatty liver disease and eventually cirrhosis. Insulin resistance is one of the characteristics of type 2 diabetes. Several types of treatment have been employed against type 2 diabetes some of which ameliorate NAFLD. The frequent line of treatment to improve insulin sensitivity is the use of thiazolidinediones (TZD) which activate the nuclear receptor, peroxisome proliferator activated receptor gamma (*Ppar*γ**). Although TZDs are proven to be very effective in promoting insulin sensitivity, its actions on *Ppar*γ** have been complicated, specifically on NAFLD. According to studies in different models, *Ppar*γ** manifests both beneficial and undesirable effects on NAFLD. This paper will focus on the current knowledge of *Ppar*γ** and its effect on NAFLD.

## 1. Introduction

Hepatic steatosis without excessive alcohol intake, called nonalcoholic fatty liver disease (NAFLD), is commonly associated with obesity and insulin resistance [1, 2]. NAFLD affects the general population and its incidence is linked with the epidemics of obesity and type 2 diabetes [3]. The metabolic pathways leading to hepatic steatosis include enhanced nonesterified fatty acid release from the adipose tissue, increased *de novo* lipogenesis, decreased *β*-oxidation and reduced VLDL export [4, 5]. Steatosis in the liver is characterized by a large intracytoplasmic fat droplet or well-defined droplets displacing the nucleus to the cell periphery [6]. The accumulation of hepatic lipids could be due to elevated peripheral fatty acids, *de novo* lipogenesis and defective apolipoprotein biosynthesis [7, 8]. The progression of hepatic steatosis often leads to liver inflammation or steatohepatitis and, if unchecked, will worsen liver fibrosis and cirrhosis [9]. Several mouse models are used to elucidate the mechanisms of fatty liver disease [10, 11]. The bulk of studies on NAFLD have been through the administration of high fat diets or methionine-choline-deficient diets and the use of genetically leptin-deficient (*ob/ob*) or leptin-receptor-deficient (*db/db*) mouse models [12]. These models exhibit insulin resistance which is one sequelae of NAFLD [13]. Increased insulin secretion has been directly implicated in the development of fatty liver disease [14]. The therapy used for insulin resistance frequently include the administration of TZDs which are agonists for the nuclear receptor peroxisome proliferator activated receptor gamma (*Ppar*γ**) [15]. However, studies have demonstrated that TZDs have also exhibited deleterious side effects that warrant their withdrawal from the market [16]. Of interest, selective *Ppar*γ** modulators (SPPARMs) that do not have the undesirable effects of TZDs have been recently identified [17, 18]. Since NAFLD often leads to insulin resistance, this paper focuses on the relationship between *Ppar*γ** and NAFLD.

## 2. Ppar-gamma *(Ppar**γ**)*

*Ppar*γ** is a member of the nuclear receptor superfamily of ligand-activated transcription factors [19] highly expressed in adipocytes [20, 21] and plays a role in improving glucose homeostasis and adipocyte differentiation [22]. It increases insulin sensitivity by upregulating the glucose transporter 4 (*Glut4*) [23]. *Ppar*γ** also enhances the transcription factors adipocyte determination and differentiation-dependent factor 1 (*Add1*) and sterol regulatory element binding protein 1 (*Srebp1*) which results in the expression of lipogenic genes such as fatty acid synthase (*Fas*) [24]. *Ppar*γ** is expressed as 2 major isoforms, **γ*1* and **γ*2*, generated from the same gene by alternate promoter usage and RNA splicing [25]. Both isoforms could stimulate adipogenesis when introduced to fibroblasts [26]. Adipocytes treated with the *Ppar*γ** ligand, TZD, stimulate expression of uncoupling protein 2 (*Ucp2*) and therefore increase energy expenditure [27]. Another mechanism of action by TZDs in the adipose tissue is to upregulate the expression of AMP-activated protein kinase (*Ampk*) which increases fatty acid oxidation while decreasing lipogenesis via downregulation of *Srebp-1c *and carbohydrate response element binding protein (*Chrebp*) [28]. The upregulation of hepatic *Ppar*γ** is frequently observed in mice fed a high fat diet [29]. In addition, liver specific deletion of *Ppar*γ** in mice established its role as a prosteatotic factor in the development of NAFLD [30]. Of importance is that *Ppar*γ** activation by TZDs promotes efflux of free fatty acids from the liver and muscle while increasing fat mass which consequently improves insulin sensitivity [31]. Therefore, whether the upregulation of* Ppar*γ** causes steatosis or vice versa remains unclear.

## 3. The Effect of *Ppar**γ*** Variants in the Development of NAFLD

Variants in the *Ppar*γ** gene found in human genotyping studies have been reported to affect hepatic steatosis. A Japanese cohort was first reported to have a polymorphism in the peroxisome proliferator activator receptor gamma coactivator 1 alpha (*Ppar*γ*c1a*) gene [32].The polymorphism in the T allele of rs2290602 was found in patients with nonalcoholic steatosis which was further confirmed by quantitative real time PCR [32]. In addition, single nucleotide polymorphisms (SNPs) in the C161T genotype in the* Ppar*γ** gene found in a Chinese population was associated NAFLD possibly through the adiponectin pathway [33]. Moreover, the Pro12Ala variant in the *Ppar*γ** gene was found to be associated with pathogenesis of NAFLD in Indian, Chinese, and North American cohorts but not in German and Italian cohorts [34–38]. Taken together, polymorphisms in the *Ppar*γ**gene could be useful to identify individuals that are at high risk for NAFLD but should not be considered as the main factor for the disease.

## 4. The Role of *Ppar**γ*** in the Development of Hepatic Steatosis

The mode of action of *Ppar*γ** in liver was suggested to promote insulin sensitivity but with concomitant development of fatty liver. High-fat diet fed mice develop hepatic steatosis and have increased *Ppar*γ** expression [39]. This could be due to the suppression cAMP response element binding protein (*Creb*) levels, the upstream regulator of *Ppar*γ**, in high fat diet fed mice [39]. In hepatic overexpression studies, Yu et al. showed that* Ppar*γ*1* leads to adipogenic hepatic steatosis [40]. This group employed the* Ppar*α** deleted (*Ppar*α**-KO) mouse model and then injected the mice with adenovirus overexpressing *Ppar*γ*1* [40]. They showed that hepatic overexpression of *Ppar*γ*1* induced adipocyte specific gene expression patterns in the livers of *Ppar*α**-KO mice [40]. Therefore, they propose that excess *Ppar*γ** activity can lead to the development of adipogenic hepatic steatosis [40]. In addition, hepatic adenoviral overexpression of *Ppar*γ*2 *in lean mice increased liver triglyceride content and induced hypertension [41]. This occurrence was reported to involve the target of *Ppar*γ**, fat specific protein 27 (Fsp27) [42] and its actions on the afferent vagal signals in the liver [41]. In a liver specific* Ppar*γ** deletion study, Gavrilova et al. reported that the A/ZIP/F-1 mouse model, which develops severe lipoatrophic diabetes, exhibited attenuation of hepatic steatosis but compromised triglyceride clearance [43]. The same group also showed that the liver *Ppar*γ** is essential for the effects of a *Ppar*γ** agonist, rosiglitazone, to improve glucose metabolism [43]. Moran-Salvador et al. reported that hepatocyte specific deletion of *Ppar*γ** in mice protected high fat diet fed mice from accumulation of lipids and, therefore, further implicated its role in the development of hepatic steatosis [30]. They also showed that the *Ppar*γ** in Kupffer cells might not be involved in the development of hepatic steatosis [30]. In addition, a mouse model of dyslipidemia showed that hepatic *Ppar*γ*2* upregulation induced hepatic *de novo *lipogenesis [44]. Zhang et al. fed a western-type diet to mice that express the human apolipoprotein B and lack the brown adipose tissue (apoB/BATless) [44]. These mice are obese, insulin resistant and have hepatic steatosis [44]. They showed that hepatic *Ppar*γ*2 *expression is increased due to elevated rates of lipogenesis via the upregulation of *de novo* lipogenic genes *Fas* and acetyl-CoA carboxylase (*Acc*) [44]. Taken together, these studies strongly implicate *Ppar*γ** in the development of hepatic steatosis.

## 5. The Role of *Ppar**γ*** in the Reduction of Hepatic Steatosis

Diet induced hepatic fibrosis mouse models that were either treated with rosiglitazone or administered with adenovirus overexpressing *Ppar*γ** were shown to ameliorate hepatic steatosis [45, 46]. Mice fed a methionine-choline-deficient (MCD) diet developed severe hepatic steatosis, inflammation, and fibrosis with downregulation of *Ppar*γ** levels [45]. Meanwhile, mice that were fed the same diet supplemented with rosiglitazone were protected from the adverse effects of the MCD diet [45]. The protection from nutritional fibrosing steatohepatitis by *Ppar*γ** could be due to the inhibition of hepatic stellate cell activation which is one of the main causes for fibrosis [45]. Similarly, the hepatic adenoviral overexpression of *Ppar*γ** in MCD diet-fed mice elicited protection from fibrotic steatohepatitis [46]. This could be explained by the genetic upregulation of adiponectin (*adipoQ*) and hemeoxygenase 1 (*Hmox1*) and the downregulation of inflammatory markers such as tumor necrosis factor alpha (*Tnf*α**) and interleukin 6 (*Il-6*) [46]. In a model of hepatic steatosis involving alcohol, mice that were fed ethanol showed amelioration of hepatic steatosis following administration of rosiglitazone due to stimulation of fatty acid oxidation in the liver [47]. In addition, the Long Evans rats, which exhibit moderate obesity and insulin resistance, were given rosiglitazone and subsequently ameliorated hepatic steatosis which could be modulated by Sirtuin 6 (*Sirt6*) and its target genes *Ppar*γ*c1a*, forkhead box protein O1 (*Foxo1*), liver kinase B1 (*Lkb1*) and 5′ adenosine monophosphate-activated protein kinase (*Ampk*) [48]. *In vitro* experiments, confirmed the function of *Sirt6* by using the free fatty acid stimulated mouse hepatocyte cell line, AML 12, which also showed the protection from hepatic steatosis following rosiglitazone treatment [48]. Similarly, Sprague-Dawley rats that were given high sucrose and high fat diet showed amelioration of hepatic steatosis following treatment with rosiglitazone [49]. The decrease in liver triglycerides in these rats could be due to the effect the *Ppar*γ** agonist in increasing serum adiponectin and the upregulation of fatty acid oxidation genes, carnitine palmitoyl transferase 1 (*Cpt1*) and acyl coenzyme A oxidase (*Aco*) [49]. Furthermore, dietary methionine restriction (MR) in F344 rats upregulated hepatic *Ppar*γ** expression, improved insulin sensitivity, and increased fatty acid oxidation [50–52]. Overall, these sets of data suggest that *Ppar*γ** ameliorated hepatic steatosis due to increased fatty acid oxidation.

## 6. The Development of Selective *Ppar**γ*** Modulators (SPPARMS)

Although* Ppar*γ** agonists have direct actions to improve insulin sensitivity, this line of treatment also has undesirable side effects. For example, rosiglitazone was reported to reduce bone mass in mice [53]. In addition, mice that are obese and diabetic develop hepatic steatosis following treatment with TZDs [54]. More recently, a meta-analysis of type 2 diabetes patients showed that pioglitazone is associated with increased risk for urinary bladder cancer [55]. The development of SPPARMS could potentially reduce these negative effects. Modifications in the *Ppar*γ** ligands showed direct effects on insulin sensitivity but not on adipogenesis [56, 57]. The ligand FMOC-L-Leucine, a chemically distinct ligand for *Ppar*γ*,* was reported to improve insulin sensitivity but did not affect hepatic lipid metabolism in *db/db* mice [58]. Telmisartan, an angiotensin receptor blocker that acts as a *Ppar*γ** ligand, enhanced insulin sensitivity and decreased body fat in high fat diet fed mice [59]. A synthetic *Ppar*γ** ligand, nTZDpa, ameliorated fasting hyperglycemia and hyperinsulinemia and caused decrease in weight gain and adipose tissue size in high fat diet fed mice [60]. In addition, results using a gene expression-based screening identified N-acetylfarnesylcysteine (AFC) as a full and partial agonist of *Ppar*γ** [61]. The compound upregulated *Ppar*γ** agonist target genes adipose differentiation-related protein (*Adrp*), angiopoietin-related protein 4 (*Angptl4*) and *adipoq*, but was only a partial agonist of adipocyte fatty acid binding protein 2 (*ap2*) [61]. The AFC also improved glucose homeostasis and reduced adipose tissue inflammation and expansion in diet-induced obese mice [61]. Furthermore, a synthetic *Ppar*γ** antagonist, SR-202, decreased expression of *Ppar*γ** target genes and promoted insulin sensitivity in diet-induced obese mice as well as in *ob/ob* mice [62]. Moreover, a partial *Ppar*γ** agonist, INT131, was designed to mitigate insulin sensitivity while minimizing the side effects of thiazolidinediones [63]. It was reported that INT131 reduced fasting plasma glucose in humans and also increased insulin sensitivity in *db/db* and diet induced obesity in mice [64, 65]. Taken together, recent data on the use of SPPARMS maintain the effects of *Pparγ* as an insulin sensitizing agent but with decreased risks for undesirable effects.

## 7. Conclusion

With all these data surrounding the effect of *Ppar*γ** on the development of NAFLD and improvements in insulin sensitivity in several models, it is still not conclusive as to whether the nuclear receptor is beneficial or detrimental. Therefore, further investigations are necessary to elucidate the effect of specific conformational and structural differences between the nuclear receptor and its ligands. The advent of the development of SPPARMS points to the direction of specifically eliciting the desirable effects of *Ppar*γ** activation.

## References

[1] Browning JD, Szczepaniak LS, Dobbins R (2004). Prevalence of hepatic steatosis in an urban population in the United States: impact of ethnicity. *Hepatology*.

[2] Yki-Jarvinen H (2005). Fat in the liver and insulin resistance. *Annals of Medicine*.

[3] Williams CD, Stengel J, Asike MI (2011). Prevalence of nonalcoholic fatty liver disease and nonalcoholic steatohepatitis among a largely middle-aged population utilizing ultrasound and liver biopsy: a prospective study. *Gastroenterology*.

[4] Postic C, Girard J (2008). The role of the lipogenic pathway in the development of hepatic steatosis. *Diabetes & Metabolism*.

[5] Postic C, Girard J (2008). Contribution of de novo fatty acid synthesis to hepatic steatosis and insulin resistance: lessons from genetically engineered mice. *Journal of Clinical Investigation*.

[6] Brunt EM, Tiniakos DG (2010). Histopathology of nonalcoholic fatty liver disease. *World Journal of Gastroenterology*.

[7] Donnelly KL, Smith CI, Schwarzenberg SJ, Jessurun J, Boldt MD, Parks EJ (2005). Sources of fatty acids stored in liver and secreted via lipoproteins in patients with nonalcoholic fatty liver disease. *Journal of Clinical Investigation*.

[8] Charlton M, Sreekumar R, Rasmussen D, Lindor K, Nair KS (2002). Apolipoprotein synthesis in nonalcoholic steatohepatitis. *Hepatology*.

[9] Milic S, Stimac D (2012). Nonalcoholic Fatty liver disease/steatohepatitis: epidemiology, pathogenesis, clinical presentation and treatment. *Digestive Diseases*.

[10] Koteish A, Diehl AM (2001). Animal models of steatosis. *Seminars in Liver Disease*.

[11] Nagarajan P, Kumar MJM, Venkatesan R, Majundar SS, Juyal RC (2012). Genetically modified mouse models for the study of nonalcoholic fatty liver disease. *World Journal of Gastroenterology*.

[12] Anstee QM (2011). Animal models in nonalcoholic steatohepatitis research: utility and clinical translation. *Liver International*.

[13] Jou J, Choi SS, Diehl AM (2008). Mechanisms of disease progression in nonalcoholic fatty liver disease. *Seminars in Liver Disease*.

[14] Chitturi S, Abeygunasekera S, Farrell GC (2002). NASH and insulin resistance: insulin hypersecretion and specific association with the insulin resistance syndrome. *Hepatology*.

[15] Guo L, Tabrizchi R (2006). Peroxisome proliferator-activated receptor gamma as a drug target in the pathogenesis of insulin resistance. *Pharmacology and Therapeutics*.

[16] Saraf N, Sharma PK, Mondal SC, Garg VK, Singh AK (2012). Role of PPARg2 transcription factor in thiazolidinedione-induced insulin sensitization. *Journal of Pharmacy and Pharmacology*.

[17] Whitehead JP (2011). Diabetes: new conductors for the peroxisome proliferator-activated receptor gamma (PPAR*γ*) orchestra. *International Journal of Biochemistry and Cell Biology*.

[18] Choi JH, Banks AS, Estall JL (2010). Anti-diabetic drugs inhibit obesity-linked phosphorylation of PPAR*γ* 3 by Cdk5. *Nature*.

[19] Cornelius P, MacDougald OA, Lane MD (1994). Regulation of adipocyte development. *Annual Review of Nutrition*.

[20] Chawla A, Schwarz EJ, Dimaculangan DD, Lazar MA (1994). Peroxisome proliferator-activated receptor (PPAR) *γ*: adipose-predominant expression and induction early in adipocyte differentiation. *Endocrinology*.

[21] Tontonoz P, Hu E, Graves RA, Budavari AI, Spiegelman BM (1994). mPPAR*γ*2: tissue-specific regulator of an adipocyte enhancer. *Genes and Development*.

[22] Kallwitz ER, McLachlan A, Cotler SJ (2008). Role of peroxisome proliferators-activated receptors in the pathogenesis and treatment of nonalcoholic fatty liver disease. *World Journal of Gastroenterology*.

[23] Wu Z, Xie Y, Morrison RF, Bucher NLR, Farmer SR (1998). PPAR*γ* induces the insulin-dependent glucose transporter GLUT4 in the absence of C/EBP*α* during the conversion of 3T3 fibroblasts into adipocytes. *Journal of Clinical Investigation*.

[24] Kim JB, Spiegelman BM (1996). ADD1/SREBP1 promotes adipocyte differentiation and gene expression linked to fatty acid metabolism. *Genes and Development*.

[25] Fajas L, Auboeuf D, Raspé E (1997). The organization, promoter analysis, and expression of the human PPAR*γ* gene. *Journal of Biological Chemistry*.

[26] Tontonoz P, Hu E, Spiegelman BM (1994). Stimulation of adipogenesis in fibroblasts by PPAR*γ*2, a lipid-activated transcription factor. *Cell*.

[27] Camirand A, Marie V, Rabelo R, Silva JE (1998). Thiazolidinediones stimulate uncoupling protein-2 expression in cell lines representing white and brown adipose tissues and skeletal muscle. *Endocrinology*.

[28] Browning JD, Horton JD (2004). Molecular mediators of hepatic steatosis and liver injury. *Journal of Clinical Investigation*.

[29] Vidal-Puig A, Jimenez-Liñan M, Lowell BB (1996). Regulation of PPAR *γ* gene expression by nutrition and obesity in rodents. *Journal of Clinical Investigation*.

[30] Moran-Salvador E, Lopez-Parra M, Garcia-Alonso V (2011). Role for PPAR*γ* in obesity-induced hepatic steatosis as determined by hepatocyte- and macrophage-specific conditional knockouts. *FASEB Journal*.

[31] Yamauchi T, Kamon J, Waki H (2001). The mechanisms by which both heterozygous peroxisome proliferator-activated receptor *γ* (PPAR*γ*) deficiency and PPAR*γ* agonist improve insulin resistance. *Journal of Biological Chemistry*.

[32] Yoneda M, Hotta K, Nozaki Y (2008). Association between PPARGC1A polymorphisms and the occurrence of nonalcoholic fatty liver disease (NAFLD). *BMC Gastroenterology*.

[33] Hui Y, Yu-yuan L, Yu-qiang N (2008). Effect of peroxisome proliferator-activated receptors-*γ* and co-activator-1*α* genetic polymorphisms on plasma adiponectin levels and susceptibility of non-alcoholic fatty liver disease in Chinese people. *Liver International*.

[34] Dongiovanni P, Rametta R, Fracanzani AL (2010). Lack of association between peroxisome proliferator-activated receptors alpha and gamma2 polymorphisms and progressive liver damage in patients with non-alcoholic fatty liver disease: a case control study. *BMC Gastroenterology*.

[35] Gawrieh S, Marion MC, Komorowski R (2011). Genetic variation in the peroxisome proliferator activated receptor-gamma gene is associated with histologically advanced NAFLD. *Digestive Diseases and Sciences*.

[36] Gupta AC, Chaudhory AK, Sukriti (2011). Peroxisome proliferators-activated receptor *γ*2 Pro12Ala variant is associated with body mass index in non-alcoholic fatty liver disease patients. *Hepatology International*.

[37] Rey JW, Noetel A, Hardt A (2010). Pro12Ala polymorphism of the peroxisome proliferatoractivated receptor *γ*2 in patients with fatty liver diseases. *World Journal of Gastroenterology*.

[38] Yang Z, Wen J, Li Q (2012). PPARG gene Pro12Ala variant contributes to the development of non-alcoholic fatty liver in middle-aged and older Chinese population. *Molecular and Cellular Endocrinology*.

[39] Inoue M, Ohtake T, Motomura W (2005). Increased expression of PPAR*γ* in high fat diet-induced liver steatosis in mice. *Biochemical and Biophysical Research Communications*.

[40] Yu S, Matsusue K, Kashireddy P (2003). Adipocyte-specific gene expression and adipogenic steatosis in the mouse liver due to peroxisome proliferator-activated receptor *γ*1 (PPAR*γ*1) overexpression. *Journal of Biological Chemistry*.

[41] Uno K, Katagiri H, Yamada T (2006). Neuronal pathway from the liver modulates energy expenditure and systemic insulin sensitivity. *Science*.

[42] Matsusue K, Kusakabe T, Noguchi T (2008). Hepatic steatosis in leptin-deficient mice is promoted by the PPAR*γ* target gene Fsp27. *Cell Metabolism*.

[43] Gavrilova O, Haluzik M, Matsusue K (2003). Liver peroxisome proliferator-activated receptor *γ* contributes to hepatic steatosis, triglyceride clearance, and regulation of body fat mass. *Journal of Biological Chemistry*.

[44] Zhang YL, Hernandez-Ono A, Siri P (2006). Aberrant hepatic expression of PPAR*γ*2 stimulates hepatic lipogenesis in a mouse model of obesity, insulin resistance, dyslipidemia, and hepatic steatosis. *Journal of Biological Chemistry*.

[45] Nan Y-M, Fu N, Wu W-J (2009). Rosiglitazone prevents nutritional fibrosis and steatohepatitis in mice. *Scandinavian Journal of Gastroenterology*.

[46] Nan YM, Han F, Kong LB (2011). Adenovirus-mediated peroxisome proliferator activated receptor gamma overexpression prevents nutritional fibrotic steatohepatitis in mice. *Scandinavian Journal of Gastroenterology*.

[47] Sun X, Tang Y, Tan X (2012). Activation of peroxisome proliferator-activated receptor-*γ* by rosiglitazone improves lipid homeostasis at the adipose tissue-liver axis in ethanol-fed mice. *American Journal of Physiology-Gastrointestinal and Liver Physiology*.

[48] Yang SJ, Choi JM, Chae SW (2011). Activation of peroxisome proliferator-activated receptor gamma by rosiglitazone increases Sirt6 expression and ameliorates hepatic steatosis in rats. *PLoS ONE*.

[49] Berthiaume M, Laplante M, Festuccia WT, Berger JP, Thieringer R, Deshaies Y (2009). Additive action of 11beta-HSD1 inhibition and PPAR-gamma agonism on hepatic steatosis and triglyceridemia in diet-induced obese rats. *International Journal of Obesity*.

[50] Elshorbagy AK, Valdivia-Garcia M, Mattocks DAL (2011). Cysteine supplementation reverses methionine restriction effects on rat adiposity: significance of stearoyl-coenzyme a desaturase. *Journal of Lipid Research*.

[51] Malloy VL, Krajcik RA, Bailey SJ, Hristopoulos G, Plummer JD, Orentreich N (2006). Methionine restriction decreases visceral fat mass and preserves insulin action in aging male Fischer 344 rats independent of energy restriction. *Aging Cell*.

[52] Plaisance EP, Greenway FL, Boudreau A (2011). Dietary methionine restriction increases fat oxidation in obese adults with metabolic syndrome. *Journal of Clinical Endocrinology and Metabolism*.

[53] Broulik PD, Sefc L, Haluzik M (2011). Effect of PPAR-gamma agonist rosiglitazone on bone mineral density and serum adipokines in C57BL/6 male mice. *Folia Biologica*.

[54] Bedoucha M, Atzpodien E, Boelsterli UA (2001). Diabetic KKAy mice exhibit increased hepatic PPAR*γ*1 gene expression and develop hepatic steatosis upon chronic treatment with antidiabetic thiazolidinediones. *Journal of Hepatology*.

[55] Colmers IN, Bowker SL, Majumdar SR, Johnson JA Use of thiazolidinediones and the risk of bladder cancer among people with type 2 diabetes: a meta-analysis.

[56] Rangwala SM, Lazar MA (2002). The dawn of the SPPARMs?. *Sciences STKE*.

[57] Wang M, Tafuri S (2003). Modulation of PPARgamma activity with pharmaceutical agents: treatment of insulin resistance and atherosclerosis. *Journal of Cellular Biochemistry*.

[58] Rocchi S, Picard F, Vamecq J (2001). A unique PPAR*γ* ligand with potent insulin-sensitizing yet weak adipogenic activity. *Molecular Cell*.

[59] Schupp M, Clemenz M, Gineste R (2005). Molecular characterization of new selective peroxisome proliferator- activated receptor *γ* modulators with angiotensin receptor blocking activity. *Diabetes*.

[60] Berger JP, Petro AE, Macnaul KL (2003). Distinct properties and advantages of a novel peroxisome proliferator-activated protein *γ* selective modulator. *Molecular Endocrinology*.

[61] Bhalla K, Hwang BJ, Choi JH (2011). N-acetylfarnesylcysteine is a novel class of peroxisome proliferator-activated receptor *γ* ligand with partial and full agonist activity in vitro and in vivo. *Journal of Biological Chemistry*.

[62] Rieusset J, Touri F, Michalik L (2002). A new selective peroxisome proliferator-activated receptor *γ* antagonist with antiobesity and antidiabetic activity. *Molecular Endocrinology*.

[63] Higgins LS, Mantzoros CS (2008). The development of INT131 as a selective PPARgamma modulator: approach to a safer insulin sensitizer. *PPAR Research*.

[64] Lee DH, Huang H, Choi K, Mantzoros C, Kim YB (2012). Selective PPARgamma modulator INT131 normalizes insulin signaling defects and improves bone mass in diet-induced obese mice. *American Journal of Physiology-Endocrinology and Metabolism*.

[65] Dunn FL, Higgins LS, Fredrickson J, Depaoli AM (2011). Selective modulation of PPAR*γ* activity can lower plasma glucose without typical thiazolidinedione side-effects in patients with Type 2 diabetes. *Journal of Diabetes and its Complications*.

